# Acute acalculous cholecystitis caused by gallbladder metastasis due to the peritoneal dissemination of gastric cancer: A case report

**DOI:** 10.1016/j.ijscr.2021.105764

**Published:** 2021-03-17

**Authors:** Hiroaki Sugita, Risa Sato, Takahiro Araki, Toshiyuki Okuda, Tamon Miyanaga, Kenji Doden

**Affiliations:** Department of Surgery, Fukui Prefectural Hospital, 2-8-1, Yotsui, Fukui, Fukui, 910-8526, Japan

**Keywords:** AAC, acute acalculous cholecystitis, ACC, acute calculous cholecystitis, EGD, esophagogastroduodenoscopy, CT, computed tomography, PTGBD, percutaneous transhepatic gallbladder drainage, Acute acalculous cholecystitis, Peritoneal dissemination, Gastric cancer, Case report

## Abstract

**Introduction and importance:**

Acute acalculous cholecystitis (AAC) is associated with a high mortality rate. AAC caused by metastasis to the gallbladder is rare. We report a case of AAC caused by gallbladder metastasis due to the peritoneal dissemination of gastric cancer.

**Case presentation:**

An 84-year-old male visited our hospital because of epigastric pain. Ultrasonography and computed tomography revealed swelling and thickening of the gallbladder wall, but stones were not observed in the gallbladder. We performed emergency surgery with a diagnosis of acute cholecystitis. Laparoscopy revealed the presence of many nodules around the abdominal cavity including the hepatoduodenal ligament. Inflammation of Calot’s triangle was severe, so we performed subtotal cholecystectomy. We also resected one of the peritoneal nodules. Macroscopically, there were no stones in the gallbladder and histopathological examination revealed acute cholecystitis and existence of adenocarcinoma involving the subserosa of the gallbladder wall and the resected peritoneal nodule. After surgery, esophagogastroduodenoscopy revealed Borrmann type II lesions at the antrum and gastric biopsy showed adenocarcinoma. He was diagnosed with advanced gastric cancer with peritoneal dissemination. His postoperative course was good.

**Clinical discussion:**

The cases of AAC caused by gallbladder metastasis have been little reported in the literature. This case is advanced gastric cancer with peritoneal dissemination and AAC was thought to be caused by peritoneal dissemination from operative and histopathological findings. We successfully treated this rare case of AAC with laparoscopic surgery.

**Conclusion:**

Although metastasis to the gallbladder is rare, it is necessary to be aware of this possibility when treating AAC.

## Highlights

• Acute acalculous cholecystitis is rare, but life threatening disease.
• Metastasis to the gallbladder is infrequent.
• Acute acalculous cholecystitis caused by peritoneal dissemination of gastric cancer is rare and immediate treatment is essential.

## 1 Introduction

Acute acalculous cholecystitis (AAC) is an uncommon disease, which is defined as gallbladder inflammation in the absence of the gallstones obstructing the cystic duct [1]. AAC accounts for only 2–10% of the cases of acute cholecystitis, but it is associated with a higher morbidity and mortality than acute calculous cholecystitis (ACC) [2,3]. Although, various pathologies are reported to cause AAC, cancer metastasis to the gallbladder is extremely rare.

Herein, we report a case of AAC caused by gallbladder metastasis due to the peritoneal dissemination of gastric cancer. This case report is compliant with the SCARE 2020 guidelines [4].

## 2 Presentation of case

**Fig. 1:**
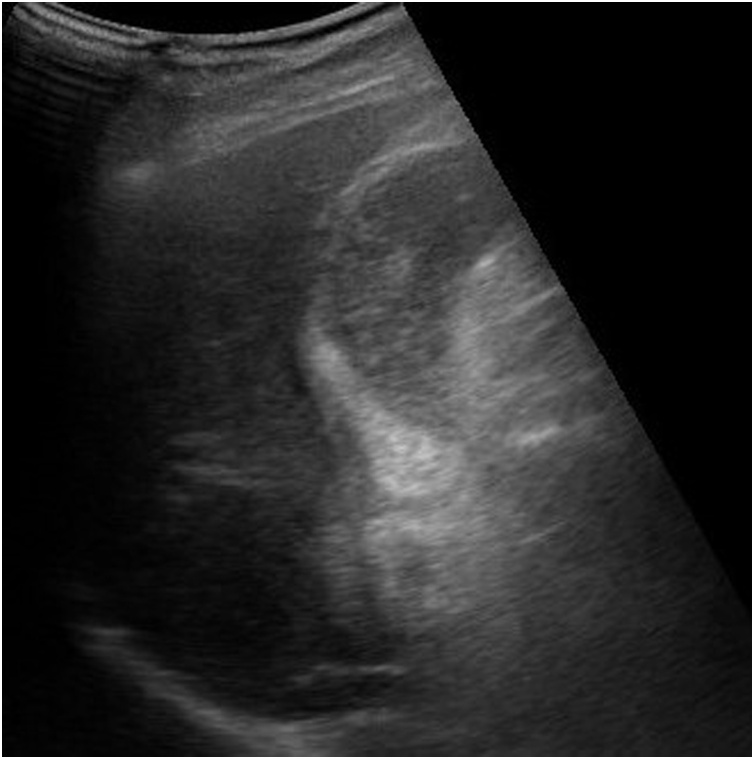
Ultrasonographic findings. Abdominal ultrasonography showed thickening of the gallbladder wall. Gallstones were not observed in the gallbladder.

**Fig. 2:**
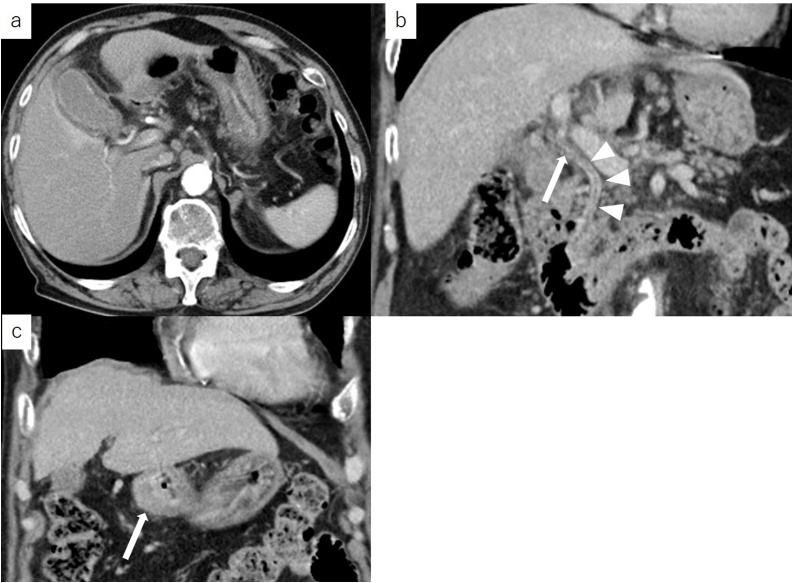
Contrast-enhanced computed tomography findings. a. Swelling and thickening of the gallbladder wall; concentration of fat in the surrounding tissue was also observed to have increased. b. There were no stones in either the cystic duct (arrow) or the extrahepatic bile ducts (arrowhead). c. Thickening of the gastric wall at the pylorus was suspected (arrow).

An 84-year-old man was transferred to our emergency center because of epigastric pain sustained from the previous day. He had hypertension as an underlying disease and he had no history of surgery. His regular medication was antihypertensive drugs. He was not a smoker and he had no history of alcohol abuse. The physical examination of the abdomen did not reveal any findings. His laboratory results showed elevated white blood cell count of 12,900/μL and a slightly elevated C-reactive protein level of 0.72 mg/dl. There was no elevation of total bilirubin and liver enzymes. Abdominal ultrasonography showed thickening of the gallbladder wall and no gallstones were observed (Fig. 1). Contrast-enhanced computed tomography (CT) revealed slight swelling and thickening of the gallbladder wall and increase in the concentration of fat in the surrounding tissue (Fig. 2a). Stones were not identified in either the gallbladder or the extrahepatic bile ducts (Fig. 2b). Thickening of the gastric wall at the pylorus was also suspected (Fig. 2c), but lymphadenopathy or distant metastases could not be identified. We performed emergency laparoscopic cholecystectomy due to the diagnosis of acute cholecystitis.

**Fig. 3:**
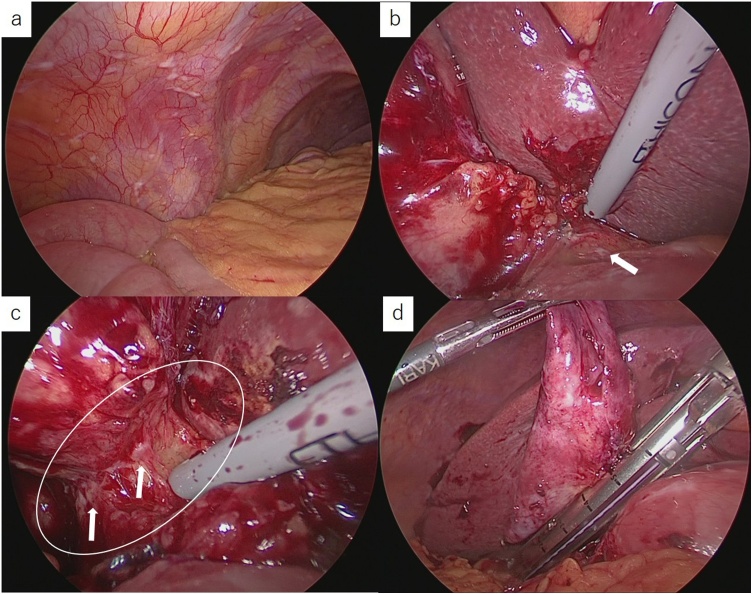
Operative findings. a.b. Many nodules were seen involving the entire abdominal cavity including the hepatoduodenal ligament (arrow). c. Severe inflammation was observed at the Calot’s triangle (circle). Some nodules were seen (arrow) and the cystic duct could not be identified. d. Neck of the gallbladder was resected using linear stapler.

Laparoscopy revealed the presence of many nodules almost involving the entire abdominal cavity including the hepatoduodenal ligament (Fig. 3a.b). The gallbladder was swollen and its wall was severely thickened. During operation, some nodules were detected at the Calot’s triangle. We could not identify the cystic duct because of severe inflammation of the Calot’s triangle (Fig. 3c); therefore, we resected the neck of the gallbladder using a linear stapler (Fig. 3d). We also resected one of the peritoneal nodules for histopathological examination. An unexposed mass at the antrum of the stomach was identified as well.

**Fig. 4:**
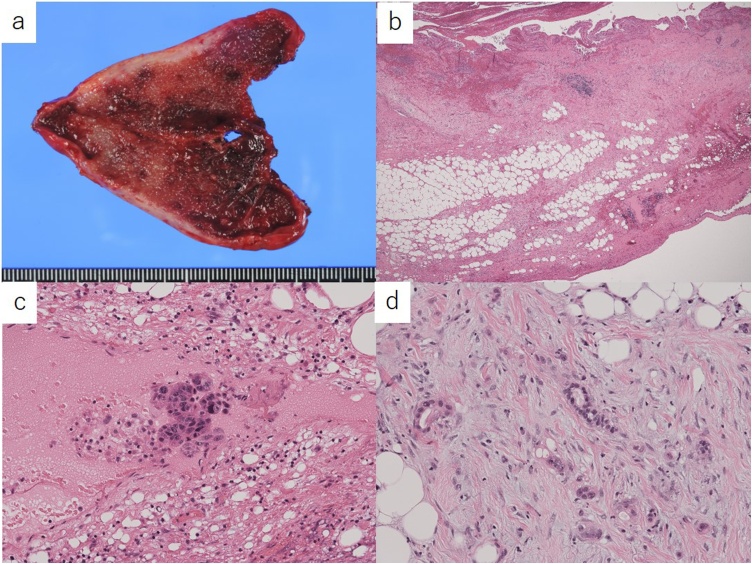
Pathological findings. a. There were no gallstones in the resected gallbladder, macroscopically. b. Histopathological examination revealed edematous thickening of the gallbladder wall. c. Moderate to poorly differentiated adenocarcinoma was detected at the subserosa of the gallbladder wall. d. A resected peritoneal nodule showed moderate to poorly differentiated adenocarcinoma.

Gross examination of the resected specimen showed that there were no gallstones in the gallbladder (Fig. 4a). Histopathological examination revealed findings of acute cholecystitis (Fig. 4b) and moderate to poorly differentiated adenocarcinoma involving the subserosa of the gallbladder wall (Fig. 4c) and the resected peritoneal nodule (Fig. 4d).

**Fig. 5:**
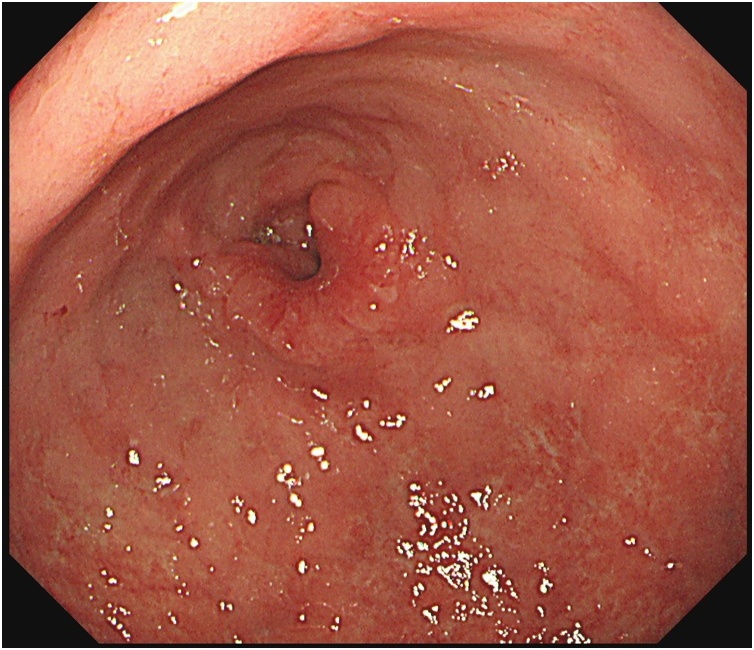
Esophagogastroduodenoscopy findings. Borrmann type II lesion was observed at the greater curvature of the gastric antrum.

Postoperative course was uneventful and he underwent esophagogastroduodenoscopy (EGD) on the third postoperative day. EGD revealed a Borrmann type II lesion on the greater curvature of the gastric antrum (Fig. 5) and gastric biopsy showed moderate to well differentiated adenocarcinoma. He was diagnosed with advanced gastric cancer with peritoneal dissemination and AAC was thought to be caused by gallbladder metastasis due to the peritoneal dissemination of gastric cancer. He was discharged on the seventh postoperative day. After that, we proposed chemotherapy for the treatment of gastric cancer, but he rejected that and requested palliative care.

## 3 Discussion

AAC usually occurs in severely injured, or critically ill patients or those patients who are under surgical stress, and comprises only 2–10% of all cases of acute cholecystitis [1,2]. AAC has a higher risk of gallbladder perforation and necrosis compared to the more typical ACC and the mortality rate associated with AAC is reported to be 30% [2,[5], [6], [7]]. If the treatment is delayed, the mortality increases to more than 50% [8], so early diagnosis and treatment are important.

The standard treatment for AAC is cholecystectomy. If the patient is critically ill or not suited for surgery because of underlying conditions, percutaneous transhepatic gallbladder drainage (PTGBD) is recommended as an alternative to surgery [9]. Treatment of AAC is essential to relieve the symptoms of the patient rapidly and to prevent the complications caused by the inflammation of gallbladder.

Metastases to the gallbladder are very rare and reported to be present in 5.8% of the patients according to the findings of a large autopsy series [10,11]. Metastases originating from malignant melanomas and lung, breast, renal, pancreatic, and colorectal cancers have been reported in the past [12,13]. In contrast, some cases of AAC were reported to be related to gallbladder cancer or cystic duct cancer, but AAC caused by the metastasis to the gallbladder is relatively infrequent. Although, a small number of AAC cases due to breast cancer metastasis and colorectal cancer metastasis have been reported [[14], [15], [16]], there are no reported cases in the literature where AAC was caused by gallbladder metastasis due to the peritoneal dissemination of gastric cancer.

In the present case, we concluded that AAC was caused by gallbladder metastasis due to the peritoneal dissemination of gastric cancer. Operative and pathological findings enabled us in diagnosing the patient with AAC caused by gallbladder metastasis. In addition to that, there were two reasons which supported our conclusion. One is that there were no typical risk factors which caused AAC such as recent surgery, trauma, severe hypovolemia, immunodeficiency, and so on. The another is that the patient had been fine before the onset of AAC.

We think that metastasis to the gallbladder and the involvement of hepatoduodenal ligament including Calot’s triangle due to the peritoneal dissemination caused severe inflammation and formation of adhesions in the Calot’s triangle, which, subsequently, resulted in AAC. Although, the wall thickness of the stomach was suspected to be increased according to the CT findings; it was difficult to identify peritoneal dissemination preoperatively using this imaging modality especially for the diagnosis of AAC.

Fortunately, we were able to perform surgery urgently for AAC without the preoperative diagnosis and the patient had a good postoperative course. Because AAC is life-threatening disease, it is important to perform suitable treatment; cholecystectomy or PTGBD. If AAC is caused by gallbladder metastasis, cholecystectomy is more suitable because of diagnosis and treatment.

AAC caused by peritoneal dissemination of gastric cancer like this case is very rare and preoperative diagnosis is challenging. We think that it is important to bear in mind the possibility of metastasis to the gallbladder as a cause of AAC and we need to perform surgery for diagnosis and treatment.

## 4 Conclusion

In conclusion, we experienced a rare case of AAC caused by gallbladder metastasis due to the peritoneal dissemination of gastric cancer. Although metastasis to the gallbladder is infrequent, it is necessary to consider its possibility as a cause of AAC.

## Declaration of Competing Interest

The authors declare that they have no conflicts of interest.

## Funding

The authors declare that they did not receive any specific funding support for this report.

## Ethical approval

No ethical approval was necessary.

## Consent

Written informed consent was obtained from the patient for publication of this case report and accompanying images. A copy of the written consent is available for review by the Editor-in-Chief of this journal on request.

## Author contribution

HS described and designed this paper. TO, TA and RS performed operation. TM, TO and RS managed postoperative care. All authors read and approved the final manuscript.

## Registration of research studies

Not applicable.

## Guarantor

Hiroaki Sugita and Toshiyuki Okuda are guarantors.

## Provenance and peer review

Not commissioned, externally peer-reviewed.
